# A Biodegradable Stereo-Complexed Poly (Lactic Acid) Drinking Straw of High Heat Resistance and Performance

**DOI:** 10.3390/ma16062438

**Published:** 2023-03-18

**Authors:** Renzhi Li, Yangyang Feng, R. Hugh Gong, Constantinos Soutis

**Affiliations:** Department of Materials, The University of Manchester, Manchester M13 9PL, UK

**Keywords:** poly (lactic acid), stereocomplex crystallites, biodegradable straws, solution casting

## Abstract

Current biodegradable drinking straws suffer from poor heat resistance and rigidity when wet, causing user dissatisfaction. Here, a fully biodegradable straw formed by stereocomplexation of poly (lactic acid) (SC-PLA) is reported. Because of the unique strong interaction and high density of link chains between stereocomplex crystallites (over 70% crystallinity), SC-PLA straws outperform their counterparts on the market. This coupled with the advantages of simple processing (solution casting and annealing) and relatively low cost (~2.06 cents per straw) makes SC-PLA drinking straws a superior substitute for plastic ones. Commercially available PLLA straws lose almost 60% of their flexural strength when wet compared to less than 5% of the SC-PLA straws proposed in this study.

## 1. Introduction

Plastics have been used widely in almost all products, from food packaging and sports equipment to vehicles, aeroplanes, and other transportation sectors. It is favoured by a wide range of industries because of its low density, high durability, low cost, and easy design and processing [1]. This high demand has naturally stimulated the mass production of plastics, but it has also raised public awareness of environmental protection, as almost all commonly used plastics are non-biodegradable, and only 9% of them are recycled [2]. Once in a landfill, they break down into tiny particles that accumulate in the natural environment [3], polluting land and waters. Long-term accumulation on land or in the ocean can have a negative impact on the health of wildlife and human health [4,5].

Plastic drinking straws are responsible for a considerable amount of plastic waste, as they are single-use and are produced and consumed in huge quantities. As a result, large corporations, such as Starbucks and McDonald’s, have introduced alternative materials to limit the use of plastic straws. Paper straws and poly (lactic acid) (PLA) straws are now widely used as environmentally friendly straws; however, paper straws lose strength properties when exposed to typical beverages [6]. In addition, they require adhesives and hydrophobic coatings to compensate for poor water resistance, which increases costs [7]. As for PLA straws, one of their major shortcomings is their poor heat resistance, i.e., PLA straws soften at temperatures around 60 °C [8], so they cannot be used for hot drinks. There is therefore an urgent need for a biodegradable straw with adequate mechanical performance and better heat resistance to replace plastic straws.

A new strategy for the development of starch straws was presented by He et al. [9]. Starch was first processed into semi-finished straws using a twin-screw extruder with a straw die; the straws were then immersed in water to adjust their moisture content and kept at 4 °C for 6 h to retrograde the starch. Finally, the straws were cross-linked using sodium trimetaphosphate (STMP). The thermal stability of the resulting straws improved due to the STMP cross-linking. Furthermore, after prolonged exposure to soft drinks, the cross-linked straws exhibited higher stiffness and final breaking force compared to the commercial rice straws currently available on the market. The authors attributed this to the possibility that STMP increased the amount of cross-linking between the starch molecules, which improved the bonding between the starch chains and provided a denser and stronger structure in the starch straws.

Yang et al. [7] prepared edible bacterial cellulose (BC)-based straws. They designed a 3D nanofibre network by introducing sodium alginate into the BC films without requiring extra adhesives. Due to the multilayer structure with the network and strong interlayer connections, the prepared straws had more than twice the strength, modulus, and toughness of paper straws. Interestingly, the edible nature of this type of straw offers another option and possibility for straws at the end of their life. After comparing the mechanical properties, biodegradability, and cost of BC-based straws with plastic and paper straws, BC-based straws proved to be a promising alternative to plastic straws.

A full cellulose straw composed of a mixture of micro- and nano-cellulose fibres was developed by Wang et al. [10]. To produce the straws, micro- and nano-cellulose fibres are first made into a wet film, which is then rolled onto a polyethylene terephthalate (PET) rod. The straws are spontaneously sealed and do not need additional binders because of the internal hydrogen bonding formed between the cellulose fibres. The formation of substantive, dense, and strong hydrogen bonds between the cellulose hybrid structures gives the straws high mechanical durability and water stability. This group [11] also reported on cellulose-lignin reinforced composite straws. The straws were produced by uniformly combining lignin powders with cellulose micro- and nanofibres to create a wet film which was then rolled up and baked. The lignin melted and penetrated the micro-nanocellulose network during baking, acting as a polyphenol adhesive to enhance the mechanical strength and hydrophobic properties of the resulting straws. Unfortunately, there are few reports on improving the poor heat resistance of PLA straws.

Due to its rich raw material sources and being completely biodegradable and biocompatible, PLA is considered one of the most promising biodegradable polymers [12,13]. It is produced from renewable resources, such as corn and starch, and it can eventually decompose into water and carbon dioxide under controlled composting conditions [14,15,16]. The tensile strength and modulus of elasticity of PLA are comparable to those of PET, but PLA has better specific tensile strength and modulus than other biopolymers [12,17,18]. As a result, PLLA has been widely used in biomedical devices, food packaging, and other applications. However, because of its low softening point (60–70 °C), PLLA is not ideal for working at high temperatures [19].

Because of the chirality of lactic acid, two enantiomeric isomers, poly (L-lactic acid) (PLLA) and poly (D-lactic acid) (PDLA), are part of the PLA family [20]. In 1987, Ikada et al. [21] reported the stereocomplexation of PLA, a unique crystallite that can be formed when PLLA and PDLA are mixed in melt or solution. Due to the strong hydrogen bonding and dipole-dipole interactions between PLLA and PDLA helical chains, as well as the tight chains stacking, stereocomplex crystallites (SC) have better mechanical properties, thermal stability, and hydrolysis resistance [22,23]. For example, the melting point of homo-crystallites (HC) formed by pure PLLA or PDLA is only 170–180 °C, while that of SC can be up to 220–230 °C. This crystal structure with improved PLA properties has gained widespread interest from researchers since it was first reported.

On this basis, this work presents a new process for the preparation of biodegradable stereocomplex crystallites-PLA (SC-PLA) straws with high thermostability and performance. SC-PLA films were first prepared by solution casting and subsequently annealed to form the straw shape without additional binders. Solution casting is a facile, short manufacturing process that does not require special equipment [24], so SC-PLA straws can therefore be produced on a large scale. Compared to the eco-friendly straws currently available on the market, SC-PLA straws have superior strength properties and heat resistance, making them suitable for use in hot drinks (>60 °C), as well as a prospective alternative to their plastic counterparts.

Depending on its exposed environment, PLA can biodegrade to different extents. In people or animals, PLA is hydrolysed to form soluble oligomers, which cells then metabolise. In the natural environment, it is hydrolysed to low molecular weight oligomers, which are then mineralised to CO_2_ and H_2_O by environmental microbes [25]. PLA has been proven to compost effectively within 90 days [15]. Sarasua et al. [26] proved that PLLA/PDLA solution casting films were still noncytotoxic materials using cytotoxicity assays, despite the addition of stereocomplex crystallites. It can therefore be expected that SC-PLA straws are nontoxic and safe to use. The biodegradability of SC-PLA materials has been reported to be slower than that of PLLA materials due to the strong interaction between l- and d-unit sequences, which suppresses the degradation of SC-PLA [27,28,29]. Therefore, SC-PLA straws can be recycled in contrast to current single-use biodegradable straws.

## 2. Materials and Methods

### 2.1. Materials and Manufacturing Process

PLLA with a weight-average molecular weight of 2.4 × 10^5^ g mol^−1^ and PDLA with a weight-average molecular weight of 6 × 10^4^ g mol^−1^ (purchased from Esun, Shenzhen, China) were used as received. Chloroform (CHCl_3_) was purchased from Sigma-Aldrich (Gillingham in UK).

The PLLA and PDLA pellets were dried under vacuum at 50 °C for 12 h. According to previous research [30,31], PLLA and PDLA were mixed 1:1 wt% and dissolved in chloroform to obtain the maximum ratio of stereocomplex crystallites. The completely dissolved solution was cast onto glass Petri dishes. In order to avoid rapid evaporation of the solvent, the Petri dishes were sealed with cling films and placed in a fume cupboard to stand at room temperature for 12 h. The films were dried under vacuum at 50 °C for 12 h to remove any residual chloroform. Then, the films were wound onto glass rods and annealed at 120 °C for 2 h. Pure PLLA solutions were also cast as the reference group.

The method for determining the optimal straw thickness is as follows: The wall thickness of the commercially available PLLA straws was first measured at 110 ± 8.74 μm; then, SC-PLA films of approximately the same thickness were cast and wound onto glass rods to develop SC-PLA straws of similar thickness to the commercial products.

### 2.2. Material Characterisation

To examine the morphologies of the films and straws, scanning electron microscopy (SEM) was used. Images of the samples were obtained using an Ultra 55 (Zeiss, Oberkochen in Germany) after gold coating.

The films and straws were scanned via an X’Pert Pro’s X-ray diffractometer (XRD) (Panalytical, Malvern in UK) with reflection mode Cu Kα radiation (voltage 40 kV, current 40 mA) at a scan rate of 2°/min and a 2θ range of 5° to 35°.

Differential scanning calorimetry (DSC) was measured using Q100 (TA instrument Ltd., Newcastle, DE, USA) in heat-cool-heat mode, with a heating rate of 10 °C/min from 35 °C to 250 °C, cooling at a rate of 10 °C/min, and heating again at a rate of 10 °C/min. Each set of samples was tested three times. The formula for calculating the crystallinity of the SC-PLA films and straws is as follows [32]:

(1) $$\;X_{c}\left(\%\right)=\frac{\Delta H_{m1}+\Delta H_{m2}-\Delta H_{c}}{\Delta H_{m\left(\mathrm{blend}\right)}^{0}}\times 100\%$$

where $\Delta H_{m1}$ and $H_{m2}$ are the melting enthalpies of the homo-crystallites (HC) and stereocomplex crystallites (SC), respectively; $\Delta H_{c}$ is the cold crystalline enthalpy; and $\Delta H_{m\left(\mathrm{blend}\right)}^{0}$ is the theoretical value for the melting enthalpy of 100% perfect HC ($\Delta H_{m1}^{0}$ = 106 J/g) and 100% perfect SC ($\Delta H_{m2}^{0}$= 142 J/g) [33,34,35], which varies with the mixing ratio of the two crystals. It can be measured as follows:

(2) $$\Delta H_{m\left(\mathrm{blend}\right)}^{0}=\Delta H_{m1}^{0}X_{h}+\Delta H_{m2}^{0}X_{s}$$

where $X_{h}$ and $X_{s}$ are the respective relative contents of HC and SC, which can be calculated as follows:

(3a) $$\begin{array}{c} \;X_{h}\left(\%\right)=\frac{\Delta H_{m1}}{\Delta H_{m1}+\Delta H_{m2}}\times 100\% \end{array}$$

(3b) $$\;X_{s}\left(\%\right)=\frac{\Delta H_{m2}}{\Delta H_{m1}+\Delta H_{m2}}\times 100\%$$

Fourier-transform infrared spectroscopy (FTIR) was used to detect changes in the functional groups of samples. Samples were cut into circles of approximately 1 cm radius and were tested on Alpha (Bruker, Coventry, UK) with a scan range of 400 to 3500 cm^−1^.

Samples were cut into 5 cm × 30 cm strips and made into paper windows for tensile testing. The mechanical tests were performed using INSTRON 3344 (Norwood, MA, USA) at a displacement rate of 5 mm/min. For the three-point bending test, the films and straws were cut into 5 cm × 20 cm strips. The support span was 10 mm, and a concentrated load was applied at the centre of the samples with a test rate of 2 mm/min. At least five specimens were tested for each material system. The flexural stress and strain of the samples can be calculated from the following equations:

(4a) $$\sigma_{f}=\frac{3\times F\times l}{2\times w\times t^{2}}$$

(4b) $$\epsilon_{f}=\frac{D\times t\times 6}{l^{2}}$$

where F is the applied force, l is the support span, w is the width of samples, t is the thickness of samples, and D is the displacement.

The thicknesses of the films and straws were measured using a thickness gauge (Mitutoyo, Kawasaki in Japan). Each sample was measured ten times at different locations and finally averaged.

In vitro hydrolysis tests were performed to investigate the degradation rate of the samples. The test samples were first weighed at their original weight and subsequently immersed in phosphate buffered saline (PBS) in a constant temperature water bath at 37 °C. PBS was changed once a week, and the samples were removed from the water bath once a week, washed with deionised water, and dried for 12 h at 50 °C in a vacuum oven to remove the moisture. The samples were then weighed again and recorded. The remaining weight of the samples is calculated as follows:

(5) $$W_{R}=\frac{W_{0}-W_{x}}{W_{0}}\times 100\%$$

where $W_{R}$ is the remaining weight of the samples, $W_{0}$ is the initial weight, and $W_{x}$ is the weight of the samples after in vitro hydrolysis at week x.

## 3. Results and Discussion

### 3.1. Structure of SC-PLA Straws

**Figure 1:**
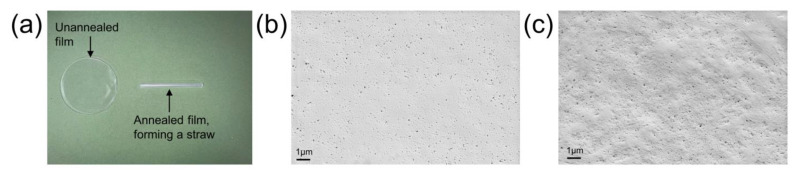
(**a**) Photo of SC-PLA film and straw. (**b**) SEM image of unannealed SC-PLA film and (**c**) SEM image of annealed SC-PLA straw.

SC-PLA straws were successfully prepared by solution casting and annealing (Figure 1a). After solution casting, a thin SC-PLA film (thickness approximately 110 μm) was produced. The film was then rolled onto a glass rod and annealed at a temperature higher than the glass transition temperature of PLA to obtain an SC-PLA straw. As the glass transition temperature of PLA is only 50–60 °C [36], annealing above this temperature causes PLA molecular chains to move freely and recrystallise, which leads to curling of the film and formation of a straw. Figure 1b,c show SEM images of SC-PLA film and straw. It can be seen that the surface of the annealed SC-PLA straw is rougher compared to the SC-PLA film. This variation in roughness may be related to the recrystallisation of molecular chains occurring in the film, which causes SC-PLA film to shrink and deform during annealing. Wang et al. [37] also reported an increase in roughness after annealing.

### 3.2. Crystallisation and Thermal Behaviour of SC-PLA Straws

**Figure 2:**
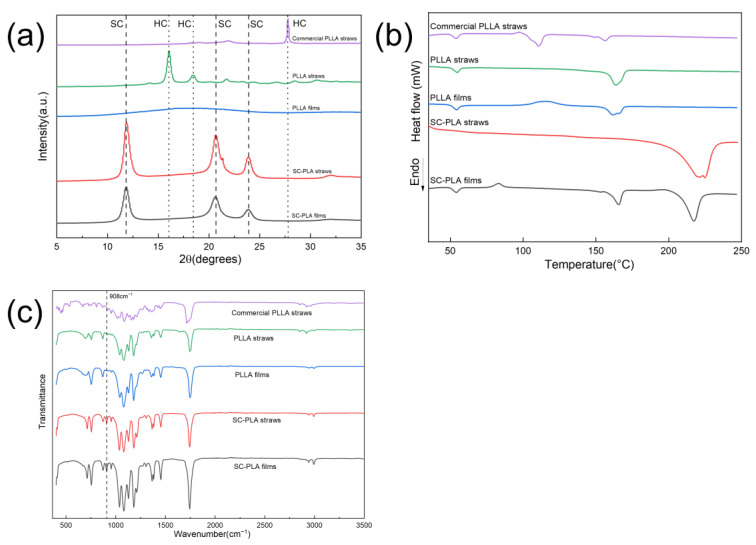
Comparison of crystallisation and thermal behaviour of films and straws. (**a**) XRD patterns, (**b**) DSC diagram, and (**c**) FTIR spectrum.

The XRD patterns of the straws are shown in Figure 2a. SC-PLA films and straws exhibit diffraction peaks at 11.8°, 20.6°, and 23.8°, corresponding to the (110), (300) and/or (030), and (220) planes of SC [38,39,40]. The diffraction peaks at 16° and 21.8° are attributed to homo-crystallites (HC), which was observed in PLLA straws. The absence of any diffraction peaks in PLLA films means that PLLA films are amorphous. After annealing (i.e., the process of transforming from films to straws), the peak areas of PLLA straws and SC-PLA straws become larger, indicating an increase in the crystallinity of the materials, which is to be expected, as the molecular chains in PLLA and SC-PLA are free to move and recrystallise during annealing. For commercial PLLA straws, only one diffraction peak is present at a 2θ value of 27.8°, which is the reflection of the β-form crystal of HC in PLLA [41,42].

Figure 2b shows the DSC diagram of the straws. The approximate 160 °C endothermic fusion peak is the melting peak for HC, while the 220 °C endothermic peak indicates the formation of SC, which is seen in SC-PLA films and straws. The disappearance of the glass transition peak and the 160 °C HC melting peak in the SC-PLA straws curve indicates (1) the high crystallinity of SC-PLA straws and (2) the absolute dominance of SC in crystallisation. Table 1 shows the DSC data for the films and straws, supporting both conclusions as well. The reason that only SC were present in the SC-PLA straws is probably attributed to the low weight-average molecular weights of the PLLA and PDLA used (2.4 × 10^5^ g mol^−1^ and 6 × 10^4^ g mol^−1^, respectively), which are important for the exclusive formation of stereocomplexation [30,43]. It is interesting to note that SC-PLA films do not exhibit any diffraction peaks of HC in XRD patterns but do show a 165.59 °C HC melting peak in the DSC diagram. The assumption is that the SC-PLA films crystallised during the heating process of DSC measurement, resulting in the formation of HC, which is reflected in the DSC curve as a cold crystallisation peak at 81.96 °C. Zhang et al. [44] also reported the production of homo-crystallites during the cold crystallisation of solution-cast films. This is also why PLLA films appear amorphous in XRD patterns but exhibit some crystallinity in DSC measurements. Furthermore, the crystallinity of PLLA and SC-PLA materials increases after annealing, which is consistent with the XRD observations. All samples except the SC-PLA straws exhibited glass transition peaks at around 54 °C, which means they will deform at temperatures above this.

The FTIR spectrum of the films and straws is shown in Figure 2c, which indicates that there was no significant structural change between the films and straws. At 908 cm^−1^, SC-PLA films and straws have a new band, which is characteristic of SC with a 3_1_ helical chain conformation, thus confirming the formation of stereocomplex crystallites [20,45,46].

### 3.3. Mechanical Properties of the SC-PLA Straws

**Figure 3:**
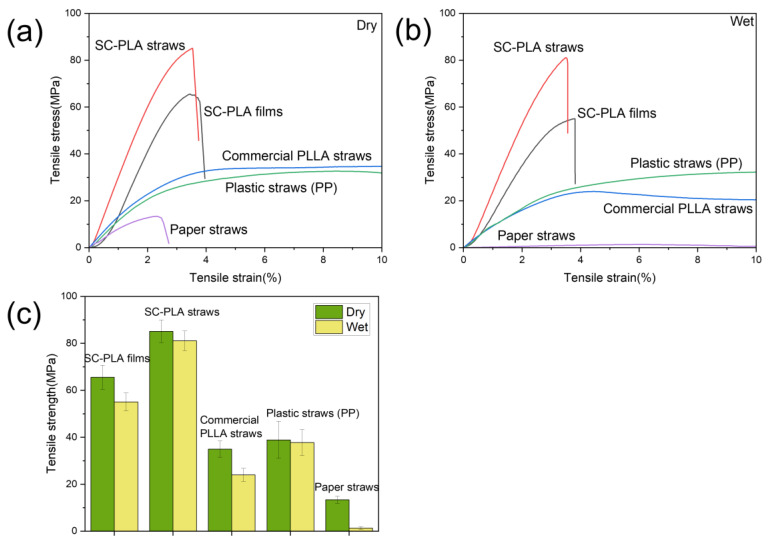
(**a**) Typical tensile stress-strain curves for all straws. (**b**) Typical wet tensile stress-strain curves for all straws immersed in hot water at 100 °C for 10 min. (**c**) Comparison of tensile strength of straws in different states.

A comparison of the tensile properties of the SC-PLA straws with other commercially available PLLA straws, plastic straws (consisting of polypropylene), and paper straws is shown in Figure 3. The procedure for the wet tensile test was to soak all straws in hot water at 100 °C for 10 minutes before performing the tensile test. The purpose of conducting wet tensile tests is to investigate the water and thermal stability of straws and to explore their potential use for hot beverages. The greater tensile strength of the SC-PLA straws compared to the SC-PLA films is due to the enhancement of the SC formed after annealing. There are two possible explanations for the enhancement effect of SC. Firstly, the formation of SC causes solutions to easily undergo three-dimensional (3D) gelation, which behaves as crosslinks as solvent evaporates. The appearance of 3D gels suggests that the linking chains between SC are highly concentrated. The outstanding mechanical performances of SC may be attributed in part to the relatively high density of linking chains. In addition, the formation of compact chain packing in the amorphous region because of strong interactions between l- and d-unit sequences may also contribute to the superior tensile strength of SC [30].

The absence of the glass transition peak in the SC-PLA straws can be seen in the DSC diagram (Figure 2b), which means that SC-PLA straws are of highly crystalline materials and that no relaxation of the molecular chains in the material occurs at 50–60 °C (the normal PLA glass transition temperature). This explains why SC-PLA straws have the lowest percentage of tensile strength loss among biodegradable straws (Table 2). This result indicates the good heat resistance of SC-PLA straws and their suitability for use with hot beverages.

**Figure 4:**
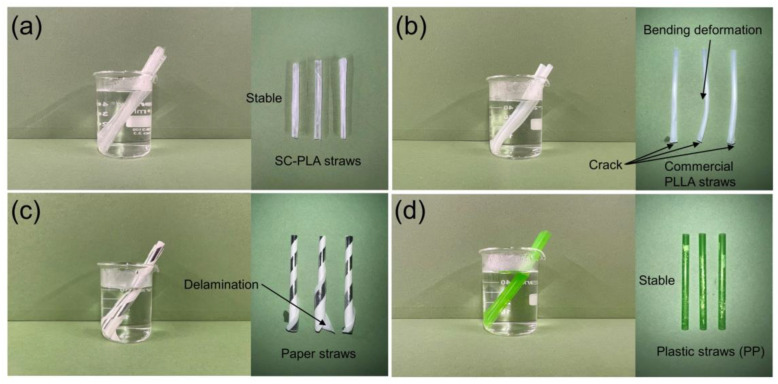
(**a**–**d**) State of three straws from each group after immersion in hot water at 100 °C for 10 min.

Figure 4a–d show the status of the respective straws after 10 min of immersion in hot water at 100 °C. The SC-PLA straw remained stable after immersion, corresponding to only a 4.71% loss of tensile strength (Table 2). This again demonstrates the high stability of SC-PLA straws in hot drinks. In contrast, a crack was observed in the commercial PLLA straw, and the entire straw was deformed. As seen from the DSC data above, this is because the glass transition temperature of commercial PLLA straws is only 54.12 °C; the straw moved from the glassy state to the highly elastic state, and the molecular chains in the straws relaxed above 54.12 °C. The paper straw showed much worse water stability. Delamination could be seen after immersion, and most of the tensile strength of the straws was lost. Unsurprisingly, the plastic straw composed of polypropylene remained stable in hot water.

**Figure 5:**
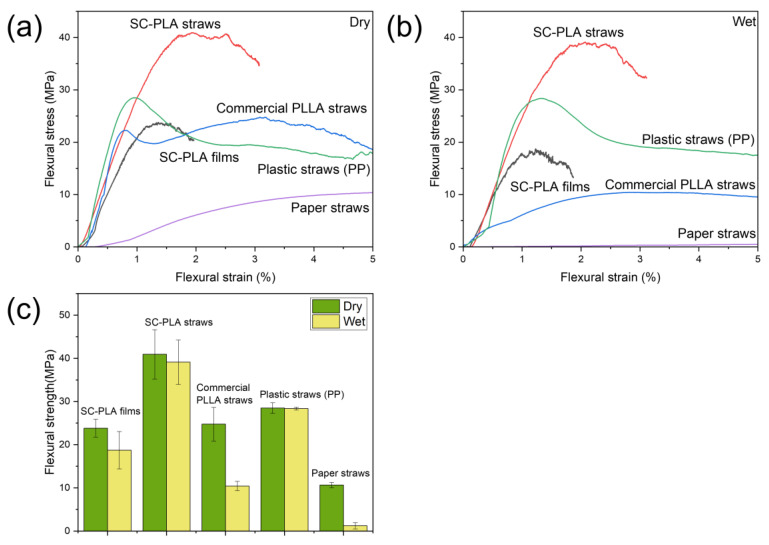
(**a**) Typical flexural stress-strain curves for all straws. (**b**) Typical wet flexural stress-strain curves for all straws immersed in hot water at 100 °C for 10 min. (**c**) Comparison of flexural strength of straws in different states.

In practice, straws are more susceptible to complex stresses, including bending [7], so three-point bending tests were performed on the SC-PLA straws and commercial straws, as shown in Figure 5. Similar to the tensile test results, the SC-PLA straws had the highest flexural strength and the least loss of flexural strength among the biodegradable straws (Table 3). This again shows that the mechanical properties of SC-PLA straws are superior to those of their commercial biodegradable counterparts.

### 3.4. In Vitro Hydrolysis of SC-PLA Straws

**Figure 6:**
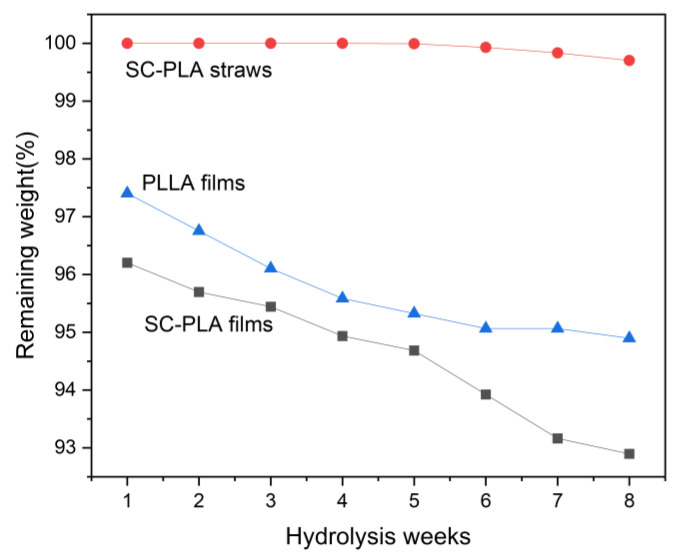
Remaining weights of films and straws as a function of hydrolysis time.

The remaining weight of the films and straws hydrolysed in vitro as a function of hydrolysis time is shown in Figure 6. The SC-PLA straws had the slowest rate of weight loss, which is mainly attributed to the increased resistance to hydrolysis introduced by SC [47,48]. As discussed in the mechanical properties section, the strong interaction between l- and d-unit sequences in the amorphous region, which delayed the diffusion of water molecules into the amorphous part of the SC-PLA straws, is suspected to be the reason for the high hydrolysis resistance of SC. The 3D micro-network structure may be another reason for the enhanced hydrolysis resistance of SC. Low molecular weight oligomers formed by hydrolysis catalyse the hydrolysis of PLA materials, and the rate of hydrolysis is proportional to the concentration of carboxyl groups in the oligomers and polymers, whereas the 3D micro-network structure reduces the concentration of catalytic oligomers produced by hydrolysis, slowing down the rate of hydrolysis of SC-PLA straws [47].

Surprisingly, the hydrolysis rate of SC-PLA films was faster than that of PLLA films, even though the XRD results showed that SC formed within the SC-PLA films, while the PLLA films were amorphous. This may be related to the difference in molecular weight between PLLA and PDLA; SC-PLA films and PLLA films were cast from the same concentration of SC-PLA solutions and PLLA solutions, respectively, but PLLA had four times the molecular weight of PDLA, resulting in more molecular chains in the PLLA films than in the SC-PLA films. Since the hydrolysis mechanism of PLA occurs by cleavage of the ester groups of the main chain [49,50], the lower molecular weight of SC-PLA films makes them less resistant to hydrolysis.

Table 4 shows that the estimated cost of SC-PLA straws is approximately 2 cents per straw, which is similar to commercial PLLA straws (approximately 2 cents per straw [10]) and less expensive than paper straws (about 4 cents per straw [10]). Therefore, SC-PLA straws are a better alternative to plastic straws because of their superior mechanical properties, thermal stability, water stability, and cost compared to commercial PLLA straws and paper straws.

## 4. Conclusions

In this work, a new process was developed which uses stereocomplex crystallites formed by PLLA and PDLA to fabricate fully biodegradable straws to address the poor thermal resistance of existing biodegradable straws. Due to the strong interaction between l- and d-unit sequences, as well as the high density of link chains between SC, the melting point of stereocomplex crystallites is increased by 50 °C, and the mechanical properties are enhanced, resulting in SC-PLA straws exhibiting improved heat resistance, tensile and flexural properties, and water stability. Combined with the advantages of relatively easy and low-cost solution casting and annealing processing, SC-PLA straws are competitive alternatives to existing plastic straws.

## Figures and Tables

**Table 1:** Average thermal values of films and straws; three tests were performed for each group.

Samples	Cold Crystallisation		Homo-Crystallites Melting		Stereocomplex Crystallites Melting		Crystallinity
	*T_c_* (°C)	Δ*H_c_* (J/g)	*T_m_*_1_ (°C)	Δ*H_m_*_1_ (J/g)	*T_m_*_2_ (°C)	Δ*H_m_*_2_ (J/g)	*X_c_* (%)
SC-PLA films	81.96 ± 1.14	6.92 ± 1.10	165.19 ± 0.63	16.90 ± 1.42	217.43 ± 0.37	62.37 ± 4.77	53.86 ± 1.93
SC-PLA straws	-	-	-	-	221.25 ± 2.53	101.30 ± 7.77	71.34 ± 5.47
PLLA films	116.24 ± 0.30	42.56 ± 5.31	162.75 ± 0.53	49.64 ± 5.09	-	-	6.68 ± 0.96
PLLA straws	-	-	164.34 ± 0.42	40.89 ± 4.15	-	-	38.58 ± 3.92
Commercial PLLA straws	96.72 ± 0.73	4.51 ± 1.07	156.46 ± 0.46	19.74 ± 1.84	-	-	14.37 ± 1.34

**Table 2:** Comparison of average tensile strength and loss of tensile strength of straws exposed to 100 °C hot water for 10 min.

Samples	Tensile Strength (MPa)	Wet Tensile Strength (MPa)	Loss of Tensile Strength (%) ^1^
SC-PLA films	65.55	55.05	16.02
SC-PLA straws	85.09	81.10	4.69
Commercial PLLA straws	34.96	23.97	31.44
Plastic straws (PP)	38.89	37.77	2.88
Paper straws	13.36	1.34	89.97

^1^ The loss of tensile strength is calculated as the difference between the tensile strength minus the wet tensile strength divided by the tensile strength.

**Table 3:** Comparison of average flexural strength and loss of flexural strength of straws exposed to 100 °C hot water for 10 min.

Samples	Flexural Strength (MPa)	Wet Flexural Strength (MPa)	Loss of Flexural Strength (%) ^1^
SC-PLA films	23.81	18.73	21.34
SC-PLA straws	40.92	39.11	4.42
Commercial PLLA straws	24.76	10.43	57.88
Plastic straws (PP)	28.49	28.37	0.42
Paper straws	10.64	1.22	88.53

^1^ The loss of flexural strength is calculated as the difference between the flexural strength minus the wet flexural strength divided by the flexural strength.

**Table 4:** Estimated cost of SC-PLA straws.

Raw Materials and Processing Cost	Approximate Usage Mass (mg/per Straw)	Approximate Cost (cents/per Straw)
PLLA	200.00	0.09
PDLA	200.00	0.17
Chloroform	7600.00	0.65
Other costs ^1^ [7]		1.15
Total		2.06

^1^ Other costs include electricity, the cost of labour, administration, general plant costs, and so on.

## Data Availability

The data presented in this study are available on request from the corresponding author.
